# Pain perception is altered in patients with medication-overuse headache but can improve after detoxification

**DOI:** 10.1186/1129-2377-14-S1-P149

**Published:** 2013-02-21

**Authors:** SB Munksgaard, L Bendtsen, RH Jensen

**Affiliations:** 1 Danish Headache Center, Dept. of Neurology, Glostrup Hospital, Faculty of Health Sciences, University of Copenhagen, Denmark

## Objective

To investigate pain perception before and during detoxification in patients with medication overuse headache (MOH).

## Background

Previously, central sensitization has been found in chronic, primary headaches but pain perception in MOH patients has only scarcely been studied and never in long-tem follow-up studies.

## Methods

35 patients with MOH following structured detoxification programmes were tested before and 2, 6 and 12 months after withdrawal and 40 age and sex matched, healthy volunteers were tested for comparison. We measured cephalic and extra cephalic pressure pain thresholds (PPT) and supra-threshold pressure pain (STPP) as well as extra cephalic pain thresholds, supra-threshold pain and wind-up for electrical stimulation.

## Results

At baseline, cephalic and extra cephalic PPTs were significantly lower in patients with MOH compared with healthy volunteers. Cephalic STPP was significantly higher in MOH patients compared with healthy volunteers but decreased significantly from baseline to the 6-month and 12-month follow-up. Supra-threshold pain for a single electrical stimulus was significantly higher in MOH patients compared with healthy volunteers. In contrast to healthy volunteers, patients with MOH did not exhibit wind-up before withdrawal. After 2 months, MOH patients had regained ability to wind-up and this persisted at 6-month and 12-month follow-up.

## Conclusions

Patients with MOH have altered pain sensation and exhibit both allodynia and hyperalgesia indicating central sensitization. Withdrawal from medication overuse causes significant decrease in central sensitization. The ability to wind-up is altered in MOH patients, probably as a consequence of medication overuse, but it can be regained after withdrawal. These findings emphasize the need for detoxification in MOH.

